# Systematic, Point-of-Care Urine Lipoarabinomannan (Alere TB-LAM) Assay for Diagnosing Tuberculosis in Severely Immunocompromised HIV-Positive Ambulatory Patients

**DOI:** 10.4269/ajtmh.19-0493

**Published:** 2020-01-20

**Authors:** Helena Huerga, Loide Cossa, Ivan Manhiça, Mathieu Bastard, Alex Telnov, Lucas Molfino, Elisabeth Sanchez-Padilla

**Affiliations:** 1 Epicentre, Paris, France;; 2 Médecins Sans Frontières, Maputo, Mozambique;; 3 National Tuberculosis Program, Ministry of Health, Maputo, Mozambique;; 4 Médecins Sans Frontières, Geneva, Switzerland

## Abstract

Point-of-care urine-lipoarabinomannan (LAM) Alere Determine TB-LAM assay has shown utility diagnosing tuberculosis (TB) in HIV-positive, severely immunocompromised, TB-symptomatic patients. We assessed LAM results in severely immunocompromised patients, who had LAM systematically performed at new or follow-up HIV consultations. This was a prospective, observational study on consecutive ambulatory, > 15-year-old HIV-positive patients with CD4 < 100 cells/µL in Mozambique. Clinical assessments and LAM were performed for all and microscopy, Xpert, sputum culture, and chest X-ray for LAM-positive participants. Patients were followed up for 6 months. Of 360 patients, half were ART-naive. Lipoarabinomannan positivity was 11.9% (43/360), higher among symptomatic patients compared with asymptomatic: 18.5% (30/162), and 6.6% (13/198), respectively, *P* = 0.001. Tuberculosis was bacteriologically confirmed in 6/35 LAM-positive patients (2 of them asymptomatic). Lipoarabinomannan positivity was associated with higher risk of mortality (adjusted odds ratio [aOR]: 4.6, 95% CI: 1.3–15.6, *P* = 0.015). Systematic urine-LAM allows for rapid TB treatment initiation in severely immunocompromised HIV ambulatory patients and identifies patients at a higher risk of death.

In 2017, there were nearly a million cases of tuberculosis (TB) among people living with HIV (PLHIV), causing 300,000 deaths.^1^ The WHO recommends symptom-based TB screening for PLHIV and further evaluation of symptomatic patients using GeneXpert MTB/RIF.^2,3^ Yet, symptom-based TB screening is not routinely performed in many HIV programs, sputum samples can be difficult to produce, and this molecular technology may not be available in low-resource peripheral health facilities. Point-of-care urine lateral flow lipoarabinomannan assay Alere Determine TB-LAM (LAM) has shown utility diagnosing TB and reducing mortality in severely immunocompromised, TB-symptomatic HIV-positive patients.^4,5^ Diagnostic accuracy studies of LAM for TB diagnosis of HIV-positive patients irrespective of signs and symptoms have provided inconsistent results.^6–13^ In a recent systematic review, the sensitivity and specificity of LAM in outpatients, irrespective of their TB symptoms, was 31% and 95%, respectively,^14^ and the just published WHO TB-LAM revised guidelines recommend the use of LAM in HIV-positive patients with CD4 count les than 100 cells/µL, irrespective of signs and symptoms of TB^15^ We assessed LAM results and TB diagnosis in ambulatory, severely immunosuppressed HIV patients, who had LAM systematically performed at new or regular HIV consultations. We also assessed the association between LAM results and mortality at 6 months.

This prospective, observational study included consecutively > 15-year-old, ambulatory HIV-positive patients with CD4 < 100 cells/µL presenting for new or follow-up HIV consultations at the Centro de Referência de Alto-Maé (CRAM), in Maputo, Mozambique, from December 2014 to November 2016. Patients taking TB treatment were not eligible. The non-provision of sputum sample was not an exclusion criterion. The CRAM is an outpatients HIV referral facility dually supported by the Mozambican Ministry of Health and Médecins Sans Frontières (MSF). The main reasons for referral to the CRAM were advanced HIV with CD4 less than 100 cells/µL, suspicion of antiretroviral therapy (ART) failure, Kaposi Sarcoma, HIV-positive drug users, hepatitis C coinfection, opportunistic infections, and adverse events associated with ART.

At the initial consultation, a clinician performed a full clinical examination that included an anamnesis and a physical examination, and a urine-LAM test (Alere Determine TB LAM Ag, Abbott, Palatine, IL [formerly Alere, Waltham, MA]). The LAM test was used at the CRAM before the study initiation. The medical staff had received a 4-hour training before using the test, and short refreshment training was given before the study initiation. During the study period, the LAM test was performed by the clinician in the same consultation room or by a nurse in a separate room. Lipoarabinomannan-positive patients were asked to provide two sputum samples (one “on-spot” and one early morning) for auramine staining and light emitting diode fluorescence microscopy, GeneXpert MTB/RIF using regular cartridges (Xpert; Cepheid, Sunnyvale, CA), and culture (BACTEC MGIT 960; BD, Franklin Lakes, NJ) GenoType *Mycobacterium* CM and GenoType *Mycobacterium* AS based on DNA STRIP technology were used to identify non-tuberculosis mycobacterium (NTM). These qualitative tests can identify the *Mycobacterium tuberculosis* complex and 27 clinically relevant NTM from liquid or solid medium culture. Chest X-ray was also requested for LAM-positive patients. Lipoarabinomannan positivity was defined as grade ≥ 1 on a 4-grade reading card. Negative LAM results required no further evaluation as per the study protocol. However, clinicians could request further TB investigations based on their clinical judgment. Lipoarabinomannan result was used to guide TB treatment. Patients were followed up for 6 months regardless of their TB treatment status.

We assessed LAM positivity, TB diagnosis, and TB treatment in the overall population. In addition, we assessed the proportion of patients with bacteriologically confirmed TB among LAM-positive patients. Finally, we assessed the proportion of patients with at least one symptom among cough, fever, weight loss, or night sweats reported in the clinical examination. The presence of any symptom was recorded, regardless of the duration and the severity. We calculated the LAM positivity among patients with at least one symptom and among patients with none of the symptoms. We also assessed the proportion of patients initiating anti-TB treatment at their first consultation, stratified by LAM result and the presence of symptoms. Finally, we assessed the association between LAM positivity and mortality at 6 months. Bacteriologically confirmed TB was defined as *Mycobacterium tuberculosis* detected by culture or Xpert MTB/RIF. Patients were considered “seriously ill” if one of the following danger signs was present: temperature > 39°C, respiratory rate > 30 respirations/minute, heart rate > 120 beats/minute, or if the patient was unable to walk without help.

Continuous variables are presented as medians with interquartile ranges (IQRs), categorical variables as counts, and percentages. We used univariate and multivariate logistic regression models with a penalized likelihood estimation to account for rare events to assess the association between LAM positivity and mortality in the first 6 months after enrollment, building age, gender, body mass index (BMI), ART, CD4 count, and the presence of TB symptoms into the models.^16^ Using a backward elimination, stepwise approach, only variables with a *P*-value < 0.2 in univariate analyses were included in the final multivariate model. Crude and adjusted odds ratios with 95% CIs were assessed, and an alpha level of 5% was used for all statistical tests. Kaplan–Meier estimates and log-rank test were used to explore difference in mortality according to LAM results. Data were analyzed using Stata v.13 (College Station, TX).

The National Ethical Review Committee of Mozambique and the MSF Ethical Review Board approved the study. Written informed consent was obtained for all patients. For those aged 16 and 17 years, consent was obtained from a parent/legal guardian and assent was obtained from the patient.

In total, 360 HIV-positive patients were included (median age 36 years, 51.1% women) (Table 1). Half were ART naive, 72.4% had CD4 < 50 cells/µL, 4.4% had a BMI < 16 kg/m^2^, and 3.6% were seriously ill. The median CD4 was 31 cells/µL [IQR: 13–53]. Overall, 198 (55.5%) did not present any symptom, 132 (36.7%) presented one symptom, and 30 (8.3%) presented two symptoms. All symptoms were mild and of short duration.

**Table 1:** Demographic and clinical characteristics at first consultation

	All, *N* = 360 *n* (%)	LAM positive, *N* = 43 *n* (%)	LAM negative, *N* = 317 *n* (%)	*P*-value
Women	184 (51.1)	20 (46.5)	164 (51.7)	0.769
Age (years), median [IQR]	36 [31–43]	37 [29–42]	36 [32–43]	0.722
BMI (kg/m^2^), median [IQR]	21 [19–24]	21 [19–23]	21 [19–24]	0.159
BMI < 16 (kg/m^2^)	16 (4.4)	5 (11.6)	11 (3.5)	0.014
Antiretroviral treatment-naïve	188 (52.2)	22 (51.2)	166 (52.4)	0.921
Seriously ill	13 (3.6)	7 (16.3)	6 (1.9)	< 0.001
Seriously ill or body mass index < 16	26 (7.2)	11 (25.6)	15 (4.7)	< 0.001
CD4 count (cells/µL)				
Median [IQR]	31 [13–53]	24 [13–46]	33 [13–56]	0.280
CD4 < 50	259 (72.4)	33 (76.7)	226 (71.8)	0.492
Reported symptoms				
Cough	64 (17.8)	12 (27.9)	52 (16.5)	0.171
Fever	32 (8.9)	25 (7.9)	7 (16.3)	0.070
Chest pain	12 (3.3)	11 (3.5)	1 (2.3)	0.695
Haemoptysis	0	0	0	–
Difficulty to breath	4 (1.1)	2 (0.6)	2 (0.7)	0.018
Night sweats	2 (0.6)	2 (0.6)	0	0.818
Weight loss	57 (15.9)	45 (14.2)	12 (28.6)	0.017
At least one of the WHO symptoms for tuberculosis screening	162 (45.0)	30 (69.8)	132 (41.6)	0.001
Clinical examination findings				
Temperature > 37.4°C	16 (4.4)	6 (13.9)	10 (3.2)	0.001
Respiratory rate > 20/minutes	146 (40.6)	28 (65.1)	118 (37.3)	< 0.001
Heart rate > 100/minutes	52 (14.4)	13 (30.2)	39 (12.3)	0.002
LAM result				
No line	289 (80.3)	–	289 (91.2)	–
Fainter than grade 1	28 (7.8)	–	28 (8.8)	–
Grade 1	20 (5.6)	20 (46.5)	–	–
Grade 2	12 (3.3)	12 (27.9)	–	–
Grade 3	6 (1.7)	6 (14.0)	–	–
Grade 4	5 (1.4)	5 (11.6)	–	–

IQR = interquartile range; LAM = lipoarabinomannan. The symptoms for TB screening include cough, fever, night sweats, and weight loss. If the patient is seriously ill, the temperature is > 39°C and/or respiratory rate is > 30/minutes and/or heart rate is > 120/minutes.

Lipoarabinomannan positivity was 11.9% (43/360). Patients with CD4 < 50 cells/µL and CD4 50–99 cells/µL had similar LAM positivity rates (*P* = 0.646). Lipoarabinomannan positivity was higher in seriously ill patients than in those not seriously ill, 53.9% (7/13) and 10.4% (36/347), respectively, *P* < 0.001, and in those with a BMI < 16 kg/m^2^ than those with higher BMI, 31.3% (5/16) and 11.0% (37/337), respectively, *P* = 0.014. Of the seriously ill, or BMI < 16 kg/m^2^ LAM-positive patients, 72.7% had a LAM grade ≥ 2. Among 317 LAM-negative patients, 28 (8.8%) had a line fainter than grade 1. Lipoarabinomannan positivity was 18.5% (30/162) among patients with symptoms and 6.6% (13/198) among asymptomatic participants (*P* = 0.001) (Figure 1). Lipoarabinomannan positivity was similar in patients with one symptom (18.9%, 25/132) and in patients with two symptoms (16.7%, 5/30), *P* = 0.772. Of the symptomatic and asymptomatic LAM-positive patients, 25/30 and 10/13 received Xpert or culture results and four and two had bacteriologically confirmed TB. Overall, among 35 LAM-positive patients with Xpert or culture results, six (17.1%) had bacteriologically confirmed TB, and among 24 LAM-positive patients with culture results, five (20.8%) had NTM isolated (none with *Mycobacterium tuberculosis* mixed infection detected). However, speciation was not possible in any of these cases (isolates were not stored or did not grow on secondary culture). No cases of rifampicin resistance were detected. Of the 43 LAM-positive patients, 34 (79.1%) initiated anti-TB treatment at the first consultation.

**Figure 1.**
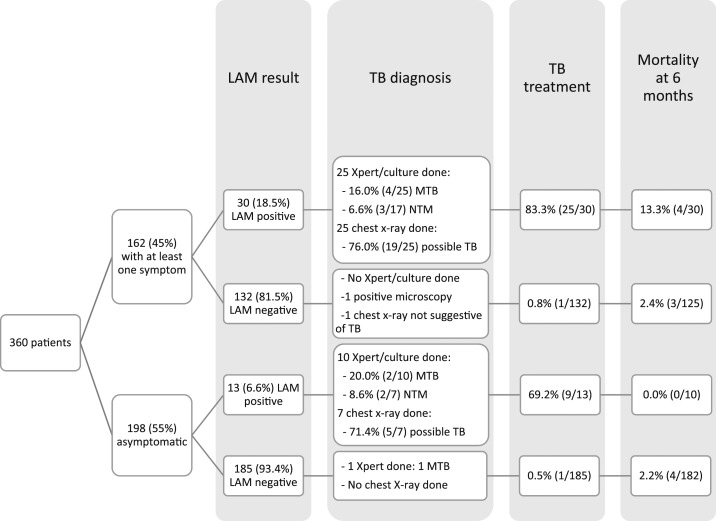
Patient flow diagram.

Overall, mortality was 3.2% (11/347). Vital status at 6 months could not be ascertained for 13 patients. Mortality was significantly higher in LAM-positive patients than in LAM-negative patients: 10.0% (4/40) versus 2.3% (7/307), log-rank test *P* = 0.009. All deceased LAM-positive patients had at least one symptom. After adjustment for CD4 count, LAM-positive results were associated with higher mortality risk (aOR: 4.6, 95% CI: 1.3–15.6, *P* = 0.015). The increased mortality risk in LAM-positive patients occurred mainly after 2 months of follow-up (Figure 2). Among LAM-negative patients, mortality was 7.1% (2/28) in patients with a visible LAM test line (fainter than grade 1) compared with 1.8% (5/278) in patients with no line, *P* = 0.071.

**Figure 2.**
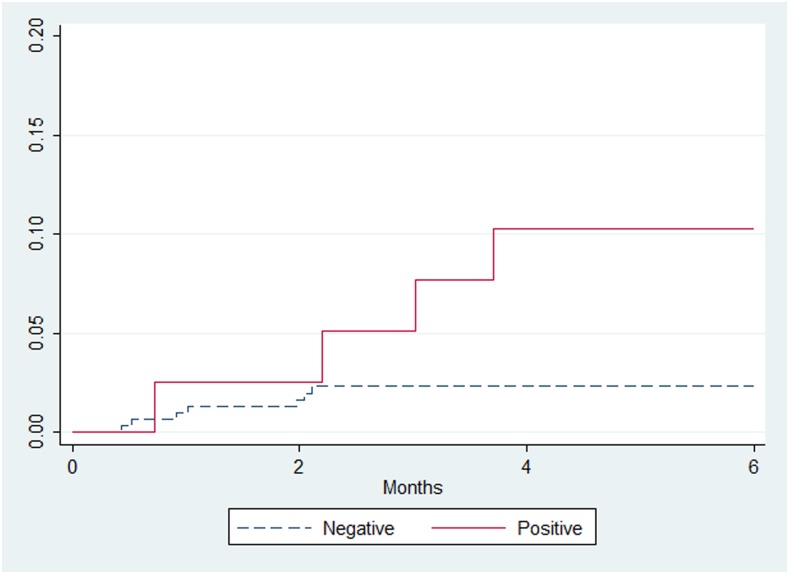
Mortality according to the lipoarabinomannan result in the first 6 months of follow-up after initial consultation among 347 patients with vital status ascertained at 6 months (log-rank test *P* = 0.009). This figure appears in color at www.ajtmh.org.

In this study, LAM positivity was relatively high among severely immunocompromised patients at new or follow-up HIV consultations. Although the large majority of the symptomatic patients had an isolated symptom and all symptoms were mild and of short duration, LAM positivity was higher in this group than the asymptomatic group. Minor symptoms may be an indication of incipient TB which underlines the importance of performing symptomatic TB screening followed by TB investigations in patients with symptoms. Systematically, using LAM allowed the immediate initiation of anti-TB treatment in 18.5% of these symptomatic patients and in 6.6% of the asymptomatic patients. These patients may have otherwise been missed.

In our study, some LAM-positive patients had negative culture results or NTM isolated. It is difficult to know whether those were false-positive LAM results or patients with TB in whom *Mycobacterium tuberculosis* did not grow in culture. Cross-reactions between LAM and NTM in patients with NTM disease have been previously reported.^17,18^ On the other hand, a positive LAM may have also been the result of an extrapulmonary or disseminated TB that the study did not assess for. Systematic LAM testing may result in the treatment of some patients who do not have TB, although LAM also identifies patients at higher risk of mortality, and the high mortality risk in severely immunosuppressed and LAM-positive patients may make this compromise worthwhile in high TB prevalence groups. In our study, the overall proportion of confirmed TB cases was low. However, some TB cases may have been missed among LAM-positive patients who were only investigated for pulmonary TB and among LAM-negative who did not receive Xpert or culture. The higher mortality among LAM-positive patients may be due to disseminated TB or could reflect immune-altered responses in these patients.^19,20^ Systematic LAM testing should not replace TB symptom screening or discourage molecular or microscopic TB investigation (especially because Xpert has higher sensitivity and detects rifampicin resistance). Rather, our results show that the combination of approaches could be an effective TB diagnosis strategy in severely immunocompromised HIV-positive patients, diagnosing those who would have otherwise been missed, allowing rapid TB treatment initiation and identifying those at higher risk of death.

Some studies of pre-ART patients have found LAM useful for diagnosing TB.^7,8,11,13^ In our study, half of the LAM-positive patients were on ART, and ART-naive patients did not have a higher likelihood of being LAM positive than those on ART, suggesting systematic LAM’s utility for diagnosing TB in severely immunosuppressed patients, regardless of their ART status. A South African study of patients under HIV care found very low LAM positivity,^6^ although high degrees of immunosuppression in our study population (median CD4 was 31 cells/µL compared with 111 cells/µL in Hanifa et al. study) may partially explain this difference.

Tuberculosis-lipoarabinomannan has practical advantages: it can be used at the point of care, it uses urine (which can be produced by almost all patients), and results are rapidly available (within 25 minutes).^21^ Implementing systematic LAM in our setting required a short training time, minimal logistical input, little extra workload for users, and was considered simple to perform.^22^ Tuberculosis is difficult to diagnose in patients with minor or subclinical symptoms, or those with extrapulmonary disease.^23–25^ Lipoarabinomannan could also help identifying TB in these patients. However, systematic urine-LAM irrespective of TB symptoms, in severely immunocompromised patients, may be complicated to implement by its dependence on CD4 measurement to select patients. Now that ART is recommended for all HIV patients and VL testing is replacing CD4 monitoring for ART patients, these cell count measures may be less available. A possible solution could be LAM-testing only patients with a higher likelihood of LAM positivity (in this study, they were those seriously ill or with low BMI), although this would mean missing a proportion of the LAM-positive patients.

This study has some limitations. It was conducted in operational research conditions where Xpert (using regular cartridge) and culture were systematically requested only for LAM-positive patients. For others, tests were performed only if requested by study or clinic staff. Therefore, we were unable to assess TB prevalence in the overall population. However, this also shows that, in practice, clinic-level TB symptom screening is often not performed properly, and symptomatic patients are often not further investigated for TB.

Systematic urine-LAM allows for rapid TB treatment initiation in severely immunocompromised, HIV-positive patients and identifies those at a higher risk of death. A diagnostic approach combining TB symptom screening and parallel LAM testing, followed by Xpert in patients with symptoms, could improve TB diagnosis in HIV-positive, severely immunocompromised, ambulatory patients. Immediate TB treatment and close medical monitoring should be ensured for LAM-positive patients. Further research is necessary to determine if this diagnostic and treatment strategy can reduce mortality in this group of patients.
